# Disabilities and Disparities in Oral Health-Related Quality of Life: A Systematic Review and Meta-Analysis in Saudi Arabia

**DOI:** 10.3390/medicina60122005

**Published:** 2024-12-04

**Authors:** Faris Yahya I. Asiri, Marc Tennant, Estie Kruger

**Affiliations:** 1 Department of Preventive Dental Sciences, College of Dentistry, King Faisal University, Al-Ahsa 31982, Saudi Arabia; 2 International Research Collaboration—Oral Health and Equity, School of Allied Health, The University of Western Australia, Perth, WA 6009, Australia; marc.tennant@uwa.edu.au (M.T.); estie.kruger@uwa.edu.au (E.K.)

**Keywords:** dental caries, disabled persons, oral health, quality of life, Saudi Arabia, systematic review

## Abstract

*Background and Objectives*: In Saudi Arabia, persons with disabilities (PWDs) face considerable oral health challenges, including a higher prevalence of dental caries and gingival inflammation, which adversely affects their oral health-related quality of life (OHRQoL). This population experiences distinct and substantial barriers in accessing adequate dental care. This systematic review and meta-analysis aims to quantify disparities in OHRQoL between PWDs and individuals without disabilities in Saudi Arabia, focusing on caries and gingivitis prevalence, and to identify specific areas for intervention. *Materials and Methods*: A structured search of PubMed, Scopus, Web of Science, and Google Scholar yielded 803 articles, of which seven met the inclusion criteria. These studies reported on OHRQoL and oral health outcomes in populations with autism, Down syndrome, cerebral palsy, and hearing impairments. Data on caries rates, gingival health, and self- or caregiver-reported quality of life were extracted and analysed. *Results*: PWDs in Saudi Arabia exhibit significantly higher caries prevalence (ranging from 60% to over 80%) and moderate-to-severe gingival inflammation (up to 60%) compared to individuals without disabilities. The caregivers of children with disabilities reported heightened stress levels, and PWDs experienced reduced functional and social well-being. These disparities were compounded by limited preventive care access and high unmet treatment needs, particularly among those with severe disabilities and limited caregiver support. *Conclusions*: PWDs in Saudi Arabia face marked oral health disparities, with notably higher rates of dental caries and gingivitis, severely impacting their quality of life. The findings underscore the need for targeted oral health policies and community-based interventions to enhance care accessibility, promote preventive measures, and address the unique needs of this vulnerable population.

## 1. Introduction

According to the World Health Organization (WHO), approximately 1.3 billion people, or about 16% of the global population, currently experience significant disability. This number is rising due to factors such as population ageing and an increase in chronic health conditions. Disability is an inherent part of the human experience, with most people likely to encounter temporary or permanent disability at some point in their lives. Persons with disabilities (PWDs) are at higher risk for conditions such as depression and poor oral health, and they face greater challenges in accessing healthcare [1]. The most common disabilities and disorders affecting oral health include intellectual and developmental disabilities, autism spectrum disorder, cerebral palsy, Down syndrome, epilepsy, and sensory impairments [2,3,4,5,6,7].

The standard of living impacts health outcomes, especially for those with disabilities or genetic conditions. Wealthier nations provide better access to prenatal testing, allowing for early detection of genetic issues, which can lead to fewer births of individuals with significant genetic disabilities due to selective pregnancy terminations [8]. Limited access to prenatal care in lower-income nations often results in higher rates of disabilities from birth [9]. Additionally, developed nations generally promote greater health awareness and provide better access to healthcare services for people with disabilities, leading to improved quality-of-life and health outcomes, such as better oral health and preventive care [10,11,12].

The caregiving environment also plays a critical role in the quality of life of individuals with disabilities. Research shows that those living alone may experience a greater sense of autonomy but face risks of isolation. Family caregiving offers emotional support but may strain caregivers, affecting care quality [13]. Specialised care facilities provide structured care with professional support, yet quality varies by resources [13]. Personal assistance programmes can support independence but rely on funding availability, so are often limited to wealthier regions [14]. The type of caregiving also impacts health; for instance, institutional care offers preventive health access [15], while family or personal care may be more individualised but with fewer resources.

Oral health is crucial to overall health and significantly impacts patients’ lives, as represented by the concept of oral health-related quality of life (OHRQoL) [16]. Traditionally, oral health has been defined as the absence of disease [17]. However, contemporary definitions recognise that oral health is multifaceted and extends beyond this definition [17]. The WHO characterises oral health as the condition of the mouth, teeth, and orofacial structures that enables individuals to carry out essential functions such as eating, breathing, and speaking. It also involves psychosocial elements such as self-esteem, overall well-being, and the ability to interact socially and work without pain, discomfort, or embarrassment [18].

Specific conditions associated with various disabilities directly affect oral health. For instance, individuals with Down syndrome are more prone to periodontal disease [19], and those with ASD may have heightened sensitivity that makes routine dental care challenging [20]. Individuals with cerebral palsy are at higher risk for occlusal problems, such as Class II malocclusion and anterior open bite [4]. Medications commonly prescribed for various disabilities can also have side effects that impact oral health, such as dry mouth, which increases the risk of dental caries and other pathologies [21]. However, PWDs often encounter barriers in accessing oral healthcare services [22], leading to poorer oral health outcomes and high unmet dental needs [23,24].

In Saudi Arabia, the prevalence of PWDs is a significant public health concern. Approximately 1 in every 30 citizens has some form of disability [25]. Recent studies have highlighted disparities in oral health outcomes and healthcare utilisation [26,27] among PWDs in the country. These findings underscore the urgent need for tailored interventions and policies to effectively address the specific oral health needs of this vulnerable population.

While the global literature provides valuable insights into the oral health disparities faced by PWDs [1,10], there remains a critical need to systematically review and synthesise existing evidence specific to Saudi Arabia. Understanding the OHRQoL among PWDs in Saudi Arabia requires a thorough assessment that considers their unique oral healthcare challenges, cultural context, and access to dental services [26,27].

A patient’s perceived health status is influenced by a multitude of factors and is not merely a reflection of their physical health. Environmental factors such as physical surroundings and social contexts, including family, friends, and coworkers, as well as personal factors such as personality traits and lifestyle, significantly shape an individual’s health perceptions [28]. The primary goal of oral healthcare is to mitigate the effects of oral diseases, focusing on improving OHRQoL. Patient-centred dental interventions aim to reduce suffering and enhance OHRQoL, ensuring that outcomes are perceived as beneficial by the patient. OHRQoL is not only crucial for individual patients but also serves as a key indicator for dental public health [29].

This review aims to address this gap by critically analysing peer-reviewed studies that investigate the OHRQoL among PWDs within the Saudi Arabian context. By understanding the extent of oral health disparities, this review seeks to provide policymakers, healthcare providers, and stakeholders with valuable insights.

## 2. Materials and Methods

### 2.1. Protocol Registration and Focused Question

A protocol was created and registered on PROSPERO prior to conducting the review (PROSPERO: CRD42024550699). Following the Participants, Exposure, Comparison, and Outcomes (PECO) framework outlined in the PRISMA guidelines [30], the following focused research question was developed: In Saudi Arabia, is the OHRQoL (**O**utcome) of PWDs (**P**articipants with **E**xposure) similar to or lower than that of persons without any disability (**C**omparison)? The PECO elements used in this review are detailed in Table 1.

The patient population included individuals in Saudi Arabia with various developmental and intellectual disabilities impacting oral health, such as cerebral palsy, autism spectrum disorder, Down syndrome, and hearing impairments. The review considered patients across a range of ages, from early childhood to adulthood, to capture a broad perspective on oral health-related quality of life (OHRQoL) among these groups. Specific inclusion criteria required that studies assess oral health using clinical indices such as the Oral Health Impact Profile (OHIP), the General Oral Health Assessment Index (GOHAI), and various quality-of-life measures, including the Child Perceptions Questionnaire (CPQ) and Parent–Caregiver Perceptions Questionnaire (P-CPQ).

### 2.2. Literature Search

#### 2.2.1. Databases and Search Strategy

The search strategy involved a structured and comprehensive search across four databases: PubMed, ISI Web of Science, Scopus, and Google Scholar. Using a combination of MeSH terms and Boolean operators, the search was tailored to include keywords related to oral health, specific disabilities, and Saudi Arabia. Specific terms included “dental health”, “oral hygiene”, and “oral health accessibility” and conditions such as “cerebral palsy”, “autism”, and “Down syndrome”. Google Scholar was included to capture grey literature and studies not indexed in the major databases. Search terms were structured using the MeSH terms and Boolean operators provided in Supplementary File S1. The initial search was performed by a medical information specialist. Screening was performed by two reviewers (FYA and EK). Any disagreements were solved by discussion. The search results were limited to 20 pages on Google Scholar to ensure manageability. 

#### 2.2.2. Inclusion Criteria

Only primary studies published in peer-reviewed journals and written in English were considered eligible. Additionally, studies had to be published within the last 25 years to ensure relevance and timeliness of the findings. These criteria aimed to focus the review on recent research that directly addressed the intersection of oral health status and quality of life among PWDs in Saudi Arabia. 

#### 2.2.3. Exclusion Criteria

Exclusion criteria ruled out studies not conducted in Saudi Arabia, non-peer-reviewed articles, interventional studies, articles not available in English, studies focusing solely on general health without specific reference to oral health, and review studies. Monthly updates of the search were repeated before the final revision of the manuscript.

#### 2.2.4. Study Selection Process

The study selection process involved initial searches in all databases, followed by title and abstract screening to identify potentially relevant studies, full-text reviews of articles meeting the initial criteria, and data extraction. All searches and reviews were performed independently by two reviewers, with discrepancies resolved through discussion or consultation with a third reviewer. The initial search was conducted on 1 July 2024 and the last update was carried out on 25 September.

### 2.3. Data Extraction

Two independent reviewers (FYA and EK) screened the titles and abstracts of retrieved records against predefined inclusion criteria. Data extraction forms were piloted and optimised before being finalised in Microsoft Excel. Studies eligible for full-text review underwent detailed assessment by the same reviewers to determine final inclusion based on relevance to the review objectives and criteria. Disagreements were resolved through consensus or by consulting a third reviewer (MT) when necessary. Data pertaining to the following categories were extracted: study name and year, study design, disabilities and relevant groups assessed, age and sex information, OHRQoL measures assessed, and reporting methods. Any outcomes related to OHRQoL were extracted for qualitative and quantitative syntheses. Any disagreements were resolved through discussion.

### 2.4. Meta-Analysis

The meta-analysis was conducted using RevMan 5.4 software, applying a random effects model to account for potential variations across the included studies. Heterogeneity among studies was evaluated using the I^2^ statistic, which quantifies the proportion of total variation due to heterogeneity rather than chance. The results of this analysis include *p*-values and 95% confidence intervals (CIs) to support statistical interpretations.

## 3. Risk of Bias

For the cross-sectional studies, JBI’s Checklist for Analytical Cross-Sectional Studies was used to assess overall bias [31]. Briefly, it includes several key domains to evaluate the methodological rigour and reliability of such studies. These domains encompass (a) a clear definition of inclusion criteria for sample selection, ensuring transparency in participant recruitment; (b) a detailed description of study subjects and settings to facilitate comparability with the target population; (c) the validation and reliability of exposure measurement methods, ensuring accuracy and consistency; (d) the use of objective criteria for measuring conditions, minimising bias in diagnostic processes; (e) the identification and acknowledgment of confounding factors that could influence study outcomes; (f) explicit strategies to address and control for confounding effects, either through study design or statistical analysis; (g) the validation and reliability of outcome measurement tools, ensuring accurate assessment of study endpoints; and (h) the appropriate application of statistical analyses tailored to study objectives and data characteristics.

Since one case–control study was identified, the JBI tool for case–control studies was used to assess the risk of bias [32]. The tool assesses case and control selection, ensuring groups are representative and well-defined to avoid misclassification or selection bias. The tool also checks if cases and controls are matched on confounding variables (e.g., age, gender) or if statistical methods control for these differences, reducing unrelated variation. Consistency in exposure measurement across groups is reviewed to prevent measurement bias, and non-respondents are considered to avoid response bias. Lastly, it examines the statistical methods used to confirm appropriate analyses for accurate interpretation of exposure–outcome relationships.

The risk-of-bias assessment was performed by two independent reviewers (FYA and EK). Disagreements were resolved through consensus or by consulting a third reviewer (MT) when necessary.

## 4. Results

### 4.1. Literature Search and Description of Included Studies

**Figure 1:**
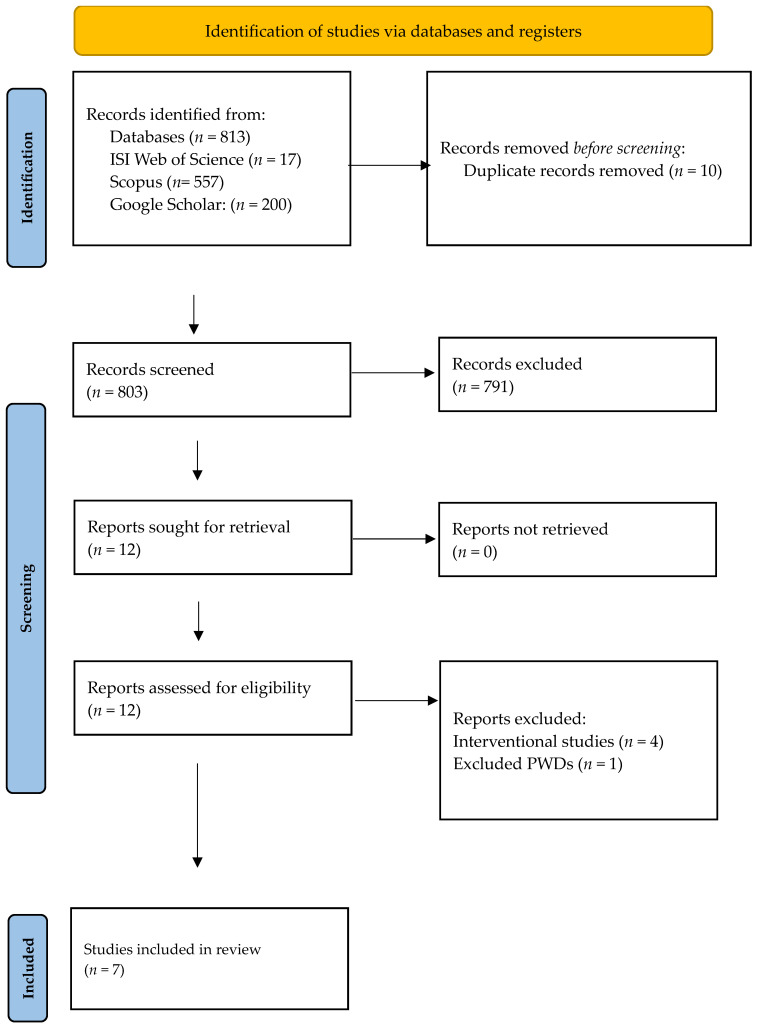
PRISMA flow diagram for study selection process.

The initial search yielded 813 records. After excluding 10 duplicates, 803 articles were screened based on their titles and abstracts. Covidence was used for management of the bibliography and duplicates were removed using the automation feature. Cohen’s Kappa for literature screening was 0.91, for data extraction it was 0.79, and for risk of bias it was 0.72. Of these, 791 articles were excluded because they did not meet the inclusion criteria for this review. As a result, 12 articles were selected for full-text review. However, five of these articles were further excluded—four due to being interventional studies [33,34,35,36] and one because it did not specifically mention or include PWDs as part of its study population [37]. Ultimately, seven articles were included in this review [38,39,40,41,42,43,44]. The results of the literature search are presented in the PRISMA flow diagram below (Figure 1).

### 4.2. Characteristics of the Included Studies

Six of the studies included in this review primarily employed cross-sectional designs [38,39,40,41,42,43]. 

Two studies focused on ASD [38,39]. The studies included in this review primarily used cross-sectional designs to explore oral health and quality-of-life outcomes among children with autism spectrum disorder (ASD) compared to neurotypical children. Pani’s study, a comparative cross-sectional study in Riyadh, involved 59 children with ASD (40 males and 17 females) and their 59 neurotypical siblings, aged 8 to 13 years. Using the parental perception questionnaire (P-CPQ) and the Family Impact Scale (FIS), this study gathered data reported by parents to assess perceived oral health quality of life [38]. Similarly, Alaki conducted a comparative cross-sectional study across ten autism centres in Jeddah, assessing 75 children with ASD (17 females, 22.7%) and 99 children without ASD (40 females, 40.4%), aged 6 to 12 years. The study utilised the Franciscan Hospital for Children Oral Health-Related Quality of Life (FHC-OHRQOL) questionnaire, also with data reported by parents, to evaluate perceived oral health impacts [39]. Both studies revealed trends in caregiver-reported oral health outcomes, emphasising challenges in maintaining oral health for children with ASD. While Pani’s study focused on the overall impact on the family using the FIS, Alaki’s study was specifically centred on the child’s quality of life as measured using FHC-OHRQOL, highlighting variations in the emphasis of quality-of-life assessments. Additionally, clinical oral health indices, such as plaque scores and decayed, missing, and filled teeth (DMFT) scores, were evaluated to provide an objective measure of oral health status in these populations.

The other studies referenced share a common focus on assessing oral health-related quality of life (OHRQoL) in children and adults with various disabilities, though they differ in terms of population characteristics, conditions, and assessment tools. Alkahtani et al.’s cross-sectional study in Riyadh assessed 146 adults (18 years and above) with hearing impairment, including 105 females and 41 males, using the General Oral Health Assessment Index (GOHAI), a self-reported questionnaire that evaluates perceived oral health quality of life [40]. Pani et al., also in Riyadh, conducted a cross-sectional study with 45 children (ages 13–17) with cerebral palsy, including 25 females and 21 males, using child and parental perception scores to assess OHRQoL [41]. Similarly, AlJameel et al. performed a cross-sectional study with 63 children (ages 10–14) with Down syndrome, including 36 females (57%) and 27 males (43%), focusing on self-reported data from the children to measure their OHRQoL [42]. AlWattban’s study in Qassim involved 107 children (ages 4–12) with multiple disabilities, using the Early Childhood Oral Health Impact Scale (A-ECOHIS) with caregiver-reported data to assess the impact of oral health on the children’s lives [43]. Finally, AlShehri et al. conducted a case–control study in Abha with 180 children, 150 with disabilities and 30 without, using the Parenting Stress Index-Short Form and the World Health Organization Quality of Life Brief Version, with data reported by both parents and children [44]. The general characteristics of the included studies are summarised in Table 2.

### 4.3. Qualitative Outcomes

#### 4.3.1. Oral Health-Related Quality of Life (OHRQoL)

Pani et al. found that siblings of autistic children scored lower in the functional limitations, emotional well-being, and social well-being domains compared to autistic children and those from non-autistic families, although no differences were observed in the oral symptoms domain. Families with autistic children had higher scores in parental emotion and family finances domains, indicating an increased emotional and financial burden [38].

Alaki et al. reported that children with autism experienced greater daily life challenges, parental concerns (*p* = 0.004 and *p* = 0.008), and poorer oral well-being (*p* = 0.000 to *p* = 0.001) than controls. Their study also highlighted that these children had higher caries prevalence (*p* = 0.013) and severity (*p* = 0.003) [39].

AlJameel et al. noted a significant impact of oral health on quality of life for both children with Down syndrome and their families. Specifically, 34.9% of children and 46% of families reported substantial impacts on quality of life, with children experiencing physical pain (54%) and families experiencing negative emotional impacts [42].

AlWattban et al. found that early childhood oral health issues significantly affected OHRQoL, especially for children with severe caries. However, the negative impact was less pronounced among children with educated caregivers or caregivers in the health sector. The average A-ECOHIS score was 10.93, and 95.3% of children were affected [43].

Alshehri et al. observed that parents of disabled children experienced significantly higher stress levels (*p* = 0.004). Factors such as dental caries severity, plaque accumulation, and caregiver education were found to significantly impact children’s OHRQoL, while BMI was not significantly related to oral health indicators [44].

#### 4.3.2. Clinical Oral Health Indices

Alkahtani et al. reported that over half of the children examined had fair oral hygiene (55.2%) and moderate gingival inflammation (60.1%). Significant differences in oral health scores were found between Saudi and non-Saudi children (1.64 vs. 1.12, *p* = 0.041), along with a high prevalence of dental caries (82.2%) and treatment needs (85.6%) [40].

Pani et al. identified that children with greater gross motor function impairments had significantly higher scores in both the child and parental perception questionnaires. Additionally, these children exhibited higher decayed, missing, and filled teeth counts (*p* = 0.002) and higher gingival index scores (*p* = 0.001) [41].

The overall outcomes of the studies are included in Table 3.

### 4.4. Risk-of-Bias Assessment Results

The quality assessment of the cross-sectional studies revealed that most adhered to the key methodological criteria. The majority of studies met the requirements for inclusion, subject selection, setting, and exposure measurement. However, there were some differences in how confounding factors were addressed. For instance, Pani [38], Alkahtani [40], and AlJameel [42] did not consider confounding factors in their analyses (Table 4).

While the case–control study by Al-Shehri et al. (2014) [44] reported the associations between plaque and dental caries and evaluated parental stress in both groups, it also relied on caregiver-reported data, which may introduce reporting bias to the overall results (Table 5).

### 4.5. Results of the Meta-Analysis

**Figure 2:**
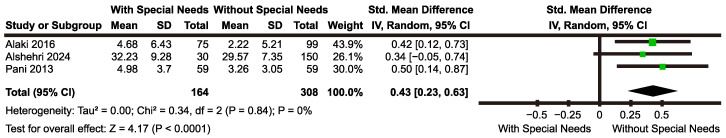
Forest plot comparing parental concern indices between parents of children with and without special needs [23,24,29].

The meta-analysis comparison of parental indices indicated there was a significantly higher concern among parents of children with disabilities. Two of the included studies focused on autism spectrum disorder (ASD), while one study did not specify the type of disabilities among the participants [23,24,29]. The forest plot reveals a combined standard mean difference (SMD) of 0.43 (95% CI: 0.23 to 0.63), indicating that parents of PWDs have a higher magnitude of stress or concerns compared to those without disabilities. The heterogeneity analysis shows an I^2^ of 0%, suggesting no observed variability between the study results. The overall effect test result is highly significant (*z* = 4.17; *p* < 0.0001), underscoring a consistent and statistically significant difference favouring the group with disabilities. Figure 2 provides a visual representation of these results.

## 5. Discussion

This systematic review examined oral health-related quality of life (OHRQoL) among persons with disabilities (PWDs) in Saudi Arabia, revealing substantial disparities in their oral health outcomes compared to their non-disabled peers. The findings indicate that PWDs—including those with autism spectrum disorder (ASD), Down syndrome, cerebral palsy, and hearing impairments—experience poorer oral health outcomes, such as a higher prevalence of dental caries, gingival inflammation, and lower overall OHRQoL [38,39,40,41,42,43,44]. These results align with broader findings from Ningrum et al., who conducted a systematic review of 20 studies and reported significant oral health inequities among Asian children with disabilities compared to their healthy peers, emphasising a regional pattern of disparities in this population [45]. Similar studies in Saudi Arabia also underscore the diminished quality of life reported by caregivers of children with disabilities, likely exacerbating these disparities by reducing caregivers’ ability to provide consistent oral care [46].

These findings underscore the need for targeted interventions to improve oral health outcomes for PWDs in Saudi Arabia. Interventional studies suggest that providing specialised dental treatments to PWDs can lead to significant improvements in OHRQoL, demonstrating the potential impact of accessible and tailored care options [33,34,35,36]. This review intends to further explore this topic in a subsequent systematic review, highlighting the importance of understanding intervention effectiveness across diverse groups.

The high rates of dental caries, gingival inflammation, and suboptimal oral hygiene observed in this population suggest that PWDs face considerable barriers to accessing and utilising oral healthcare services. For instance, elevated parental stress and lower OHRQoL scores among children with disabilities point to the broad impact that poor oral health has on both individuals and their families [38,39]. The higher parental concerns reported in these studies may also indicate increased awareness and willingness among caregivers to seek oral health interventions for their children with disabilities. However, these findings collectively suggest that current healthcare services may not be sufficiently meeting the needs of PWDs, necessitating the implementation of comprehensive strategies to improve the accessibility and quality of care.

The clinical implications of these disparities are significant. Poor oral health can adversely impact overall health and well-being, affecting essential functions such as nutrition, communication, and social interaction. For PWDs, untreated health issues can exacerbate pre-existing conditions, thereby further diminishing their quality of life and social integration [45,46]. These disparities call for action from policymakers, healthcare providers, and stakeholders to implement and support evidence-based interventions that address the unique needs of PWDs and bridge gaps in care access and quality. Targeted efforts to improve oral health outcomes for PWDs in Saudi Arabia could ultimately help reduce these disparities and enhance overall well-being for this vulnerable population.

While this review is among the few studies to evaluate the influence of OHRQoL on PWDs, providing valuable insights into oral health disparities through a comprehensive systematic review and meta-analysis in Saudi Arabia, several limitations warrant consideration. First, most of the studies included were cross-sectional, capturing data only at a single point in time and limiting insights regarding causality. Future research should incorporate longitudinal designs to better elucidate causal relationships between disability status and oral health outcomes. Additionally, the reliance on self-reported or caregiver-reported data may introduce recall bias, potentially affecting data accuracy. This limitation suggests a need for objective clinical assessments in future studies to enhance the reliability of findings. Evidence highlights the ongoing exclusion of children with disabilities from oral health research, which limits the development of strategies tailored to their unique needs and perpetuates oral health disparities [47]. Future research should prioritise inclusive methodologies that actively involve children with disabilities. By adopting such approaches, researchers can better inform the design of targeted services and policies, ultimately reducing inequalities in oral health outcomes. Additionally, this review was limited to studies published in English, which may have resulted in an incomplete picture due to excluding potentially relevant studies in other languages.

Cultural and systemic factors, such as stigma surrounding disabilities, reliance on family caregivers, and limited awareness of their specific needs, may influence access to healthcare services, potentially impacting oral health quality of life. Further research should investigate the specific impact of these factors on oral health-related quality of life to better inform targeted interventions and policies [48]. While these challenges are not unique to Saudi Arabia, they manifest differently across sociocultural and healthcare contexts [49], highlighting the importance of contextualising findings regionally while considering global relevance. The included studies varied in design, assessment tools, and sample populations, adding variability to the findings. While meta-analyses allow for some generalisation of the results, caution is suggested in applying these results universally due to the variability in assessment tools and populations. Standardised assessment methods are crucial for improving comparability across studies, thereby facilitating more robust conclusions.

In addressing these oral health disparities, it is essential to develop, test, and implement tailored interventions for PWDs. Effective interventions should prioritise accessibility, affordability, and quality of care, ensuring that PWDs receive equitable oral health services. Collaborative efforts among policymakers, healthcare providers, and community organisations are critical for creating and sustaining such initiatives. By fostering partnerships across these sectors, stakeholders can work together to promote improved oral health outcomes for PWDs, ultimately reducing health disparities and enhancing quality of life for this underserved population.

## 6. Conclusions

In conclusion, this systematic review reveals substantial disparities in oral health-related quality of life (OHRQoL) among persons with disabilities (PWDs) in Saudi Arabia. PWDs experience poorer oral health outcomes—including higher rates of dental caries, gingival inflammation, and reduced OHRQoL—compared to their non-disabled peers. These findings underscore an urgent need for targeted, accessible interventions to address these inequities and improve oral healthcare access for this vulnerable population. Addressing these disparities is essential to promoting the overall health, well-being, and quality of life of PWDs, highlighting the role of tailored healthcare strategies and collaborative support from policymakers, healthcare providers, and communities.

## Figures and Tables

**Table 1:** PECO elements used for this review.

Population	Exposure	Comparison	Outcome
Persons with disabilities (PWDs) in Saudi Arabia, including individuals with autism, Down syndrome, cerebral palsy, and hearing impairments	Disabilities and related oral health challenges	Persons without disabilities in Saudi Arabia	Oral health-related quality of life (OHRQoL), including prevalence of dental caries, gingival inflammation, and overall oral health status

**Table 2:** General characteristics of included studies assessing OHRQoL among PWDs.

Study	Study Design	Location and Setting	Group(s) with Disabilities (N)	Age and Sex	OHRQoL Measure	Method of Reporting
Pani et al., 2013 [38]	Cross-sectional (comparative)	Riyadh; setting: NR	Children with ASD (59) Siblings without ASD (59)	8–13 years Children with ASD: Males: 40; Females: 17	Parental perception questionnaire (P-CPQ) Family Impact Scale (FIS)	Proxy by parents
Alaki et al., 2016 [39]	Cross-sectional (comparative)	Jeddah; centres for autism (*n* = 10)	Children with ASD (75) Children without ASD (99)	6–12 years Females: 17 (22.7%) with ASD; 40 (40.4%) without ASD Males: NR	Franciscan Hospital for Children Oral Health–Related Quality of Life (FHC-OHRQOL) questionnaire	Proxy by parents
Alkahtani et al., 2019 [40]	Cross-sectional	Riyadh; centres: NR	Adults with hearing impairment (146)	18+ years Females: 105 Males: 41	General Oral Health Assessment Index—questionnaire	Self-reported
Pani et al., 2020 [41]	Cross-sectional	Riyadh; dental hospital (*n* = 1)	Cerebral palsy (45)	13–17 years Females: 25 Male: 21	Child perception score Parental perception score	Proxy by parents and self-reported
AlJameel et al., 2021 [42]	Cross-sectional	Riyadh; centres for disabilities (*n* = 2)	Down syndrome (63)	10–14 years Female: 36 (57%) Male: 27 (43%)	Overall OHRQoL	Self-reported
Alwattban et al., 2021 [43]	Cross-sectional	Qassim; hospital	Children with multiple disabilities (107)	4–12 years	Early Childhood Oral Health Impact Scale	Proxy by caregiver
AlShehri et al., 2024 [44]	Case–control	Abha; public schools	Children with multiple disabilities	4–14 years disabilities (*n* = 150) Without disabilities (*n* = 30)	Parenting Stress Index-Short Format World Health Organization Quality of Life Brief Version	Proxy by parents and self-reported

ASD = autism spectrum disorder; OHRQoL = oral health-related quality of life; NR = not reported.

**Table 3:** Oral health-related quality of life variables and outcomes in the reviewed studies.

Study	OHRQoL Variables Assessed	Outcomes
Pani et al., 2013 [38]	Parental–Caregiver Perceptions Questionnaire (P-CPQ) Family Impact Scale (FIS) Domains: functional limitations (FL), emotional well-being (EWB), social well-being (SWB), oral symptoms (OS)	Siblings of autistic children had significantly lower scores in the FL, EWB and SWB domains than autistic children and children from families without an autistic child (no significant differences in the OS domain). Children from families without an autistic child showed no significant difference in scores compared to parental scores for children with autism in the FL, EWB, and SWB domains. FIS scores: Families with an autistic child had higher overall FIS scores, although they were not statistically significant. Domain-specific FIS scores: Families with an autistic child had significantly higher scores in the parental emotion and family finances domains; there were no significant differences in the family activities or family conflict domains.
Alaki et al., 2016 [39]	Daily life problems Parental concerns Oral well-being Oral health status (caries, oral hygiene, gingival health, and extra-oral and intra-oral abnormalities)	Children with autism reported significantly more daily life problems and parental concerns (*p* = 0.004 and *p* = 0.008, respectively). Children with autism had significantly lower scores in oral well-being (*p* = 0.000 to *p* = 0.001). The autism group showed more extra-oral and intra-oral findings (*p* = 0.000 for both). Autism group had higher caries prevalence (*p* = 0.013) and severity (*p* = 0.003) than controls.
Alkahtani et al., 2019 [40]	General Oral Health Assessment Index (GOHAI)-Ar Oral hygiene index-simplified (OHI-S) Plaque index (PI) Gingival index (GI) Dental caries Treatment needs Decayed, missing, and filled teeth (DMFT) scores	Oral hygiene: More than half had fair OHI-S (55.2%) and PI scores (54.2%). Gingival health: 60.1% had moderate gingival inflammation. Dental caries: Prevalence of 82.2%. Treatment needs: 85.6%. Mean oral hygiene scores: Significant differences between Saudi and non-Saudi nationals ([1.64] vs. [1.12]; *p* = 0.041). Missing and filled teeth: Significant differences across different age groups (*p* = 0.000). Mean GOHAI-Ar score: 14.44 ± 9.59 (low). Spearman’s test: Significant positive correlation between GOHAI-Ar score and toothbrushing method (r = 0.164, *p* = 0.047). Negative correlations: Toothbrushing time, oral hygiene material, last visit to dentist, OHI-S score, PI score, and DMFT scores.
Pani et al., 2020 [41]	Gross Motor Function Classification Scores (GMFCS) Child perception questionnaire for adolescents (CPQ) Parental perception questionnaire (PPQ) Decayed, missing, and filled teeth (DMFT) scores Gingival index (GI)	Children with level III GMFCS had significantly higher CPQ and PPQ scores than those with level I or level II. Significant association between GMFCS and CPQ score in children (*p* = 0.016). No significant associations between CPQ score and either DMFT or GI scores. Children with GMFCS level III had significantly higher DMFT (*p* = 0.002) and GI (*p* = 0.001) scores.
AlJameel et al., 2021 [42]	Impact of oral health on quality of life of children with Down syndrome Impact of children’s oral health on family quality of life Physical pain experienced by children Emotional impact on families	34.9% of children and 46% of their families reported that their quality of life was affected by oral health. 54% of children experienced physical pain, which was severe in 22.2% of cases. Families’ emotional lives were negatively affected by children’s oral health status.
Alwattban et al., 2021 [43]	Early Childhood Oral Health Impact Scale (A-ECOHIS) The dmft/DMFT index and caries severity were determined. BMI (kg/m^2^) of the children was recorded based on height and weight measurements.	The average A-ECOHIS score was 10.93, indicating a significant impact on OHRQoL. OHRQoL was negatively affected in 95.3% of children, with the most common issue reported being ‘pain in the teeth, mouth or jaws’ (48.7%). Regression analysis indicated that children who were caries-free or had moderate caries were less likely to have a negative impact on their OHRQoL compared to those with severe caries. Caregivers with maximum primary education or working in the health sector were associated with less negative impact on children’s OHRQoL. Children ≤ 6 years old were more likely to experience a negative impact on their OHRQoL. BMI did not significantly affect the OHRQoL of the children.
AlShehri et al., 202 4 [44]	The Arabic version of the 36-item Parenting Stress Index-Short Form (PSI-SF) was used to assess parental stress. The Arabic version of the WHOQOL-BREF (World Health Organization Quality of Life Brief Version) questionnaire was used to assess the quality of life of children. Children with disabilities (certified by a paediatrician) between the ages of 4 and 14 years and their caregivers were included. Data were analysed using statistical software.	The mean PSI scale (parental stress) score was significantly higher among parents of disabled children compared to parents of healthy children (*p* = 0.004). There was no statistically significant correlation found between BMI and plaque or between BMI and DMFT + df (decayed, missing, and filled teeth) in the cases. However, there was a statistically significant correlation between plaque accumulation and DMFT + df values in the cases. The social relationship domain score in the WHOQOL-BREF questionnaire varied significantly based on parents’ educational status. Dental caries severity, plaque accumulation, and caregivers’ education level had significant impacts on the OHRQoL of children. BMI did not show a significant relationship with DMFT or plaque scores.

BMI = body mass index.

**Table 4:** Quality assessment of included studies using JBI checklist for cross-sectional studies.

Study	Criteria for Inclusion	Subjects and Setting	Exposure Measurement	Standard Criteria	Confounding Factors	Strategies for Confounding	Outcomes Measurement	Statistical Analysis
Pani et al., 2013 [38]	Yes	Yes	Yes	Yes	No	No	Yes	Yes
Alaki et al., 2016 [39]	Yes	Yes	Yes	Yes	Yes	Yes	Yes	Yes
Alkahtani et al., 2019 [40]	Yes	Yes	Yes	Yes	No	No	Yes	Yes
Pani et al., 2020 [41]	Yes	Yes	Yes	Yes	Yes	Yes	Yes	Yes
AlJameel et al., 2021 [42]	Yes	Yes	Yes	Yes	No	Yes	Yes	Yes
Alwattban et al., 2021 [43]	Yes	Yes	Yes	Yes	Yes	Yes	Yes	Yes

**Table 5:** Risk-of-bias assessment results for Al-Shehri et al. (2014) [44], using JBI checklist for case–control studies.

Criteria	Assessment
Selection of Cases and Controls	Yes
Case Definition	Yes
Control Definition	Yes
Matching/Comparability	Yes
Exposure Measurement	Yes
Same Measurement Method	Yes
Non-Response Bias	Unclear
Statistical Analysis	Yes

## Data Availability

Study data are available from the corresponding author on request.

## Supplementary Materials

The following supporting information can be downloaded at https://www.mdpi.com/article/10.3390/medicina60122005/s1: Supplementary File S1: Search terms used in the included databases.

## Abbreviations

A-ECOHIS	Early Childhood Oral Health Impact Scale
ASD	Autism spectrum disorder
BMI	Body mass index
CPQ	Child perception questionnaire for adolescents
EWB	Emotional well-being
dmft	Decayed, missing, and filled teeth (primary dentition)
DMFT	Decayed, missing, and filled teeth (permanent dentition)
GMFCS	Gross Motor Function Classification Scores
FIS	Family Impact Scale
FL	Functional limitations
GI	Gingival index
GOHAI	General Oral Health Assessment Index
JBI	Joanna Briggs Institute
OHRQOL	Oral health-related quality of life
OHI	Oral health index-simplified
OS	Oral symptoms
PECO	Participants, Exposure, Comparison, and Outcomes
PICO	Participants, Intervention, Comparison, and Outcomes
PPQ	Parental perception questionnaire
P-CPQ	Parental–Caregiver Perceptions Questionnaire
PRISMA	Preferred Reporting Items for Systematic Reviews and Meta-Analyses
PROSPERO	International Prospective Register of Systematic Reviews
PSI-SF	Parenting Stress Index-Short Form
SWB	Social well-being
PWD	Persons with disabilities
WHO	World Health Organization
WHOQOL-BREF	World Health Organization Quality of Life Brief Version
