# Supplementary material for: Disabilities and Disparities in Oral Health-Related Quality of Life: A Systematic Review and Meta-Analysis in Saudi Arabia
Source: Medicina (Kaunas). 2024 Dec 4;60(12):2005. doi: 10.3390/medicina60122005 (PMC11727891; doi:10.3390/medicina60122005)
Supplement: Supplementary file 1 [file medicina-60-02005-s001.zip › medicina-3287767-supplementary.pdf]

Total: 813

PubMED/Medline: 39 studies

((dental health OR oral healthcare OR oral health OR dental caries OR periodontal health OR periodontitis OR oral health status OR oral hygiene OR oral health measures OR cariogenic diet OR toothbrushing OR smoking OR oral health barrier OR oral health accessibility OR (oral health barrier OR barriers to oral health) OR (oral health accessibility OR access to oral health)) AND (Saudi Arabia OR Kingdom of Saudi Arabia) AND (cerebral palsy OR autism OR Asperger syndrome OR Down syndrome OR dyslexia OR dyscalculia OR dyspraxia OR dysgraphia OR blindness OR visual impairment OR deafness OR hearing loss OR hearing impairment OR Attention deficit hyperactivity disorder OR cystic fibrosis OR learning disability OR developmental disorder OR learning disorder OR intellectual disability OR autism spectrum OR hearing impaired)) AND (Oral Health-Related Quality of Life OR Oral Health Impact Profile OR General Oral Health Assessment Index OR Oral Impacts on Daily Performance OR Child-Oral Impacts on Daily Performances OR Early Childhood Oral Health Impact Scale OR Child Perceptions Questionnaire OR Parent-Caregiver Perceptions Questionnaire OR Dental Impact on Daily Living OR Oral Motor Dysfunction Questionnaire)

ISI Web of Science: 17

((dental health OR oral healthcare OR oral health OR dental caries OR periodontal health OR periodontitis OR oral health status OR oral hygiene OR oral health measures OR cariogenic diet OR toothbrushing OR smoking OR oral health barrier OR oral health accessibility OR (oral health barrier OR barriers to oral health) OR (oral health accessibility OR access to oral health)) AND (Saudi Arabia OR Kingdom of Saudi Arabia) AND (cerebral palsy OR autism OR Asperger syndrome OR Down syndrome OR dyslexia OR dyscalculia OR dyspraxia OR dysgraphia OR blindness OR visual impairment OR deafness OR hearing loss OR hearing impairment OR Attention deficit hyperactivity disorder OR cystic fibrosis OR learning disability OR developmental disorder OR learning disorder OR intellectual disability OR autism spectrum OR hearing impaired)) AND (Oral Health-Related Quality of Life OR Oral Health Impact Profile OR General Oral Health Assessment Index OR Oral Impacts on Daily Performance OR Child-Oral Impacts on Daily Performances OR Early Childhood Oral Health Impact Scale OR Child Perceptions Questionnaire OR Parent-Caregiver Perceptions Questionnaire OR Dental Impact on Daily Living OR Oral Motor Dysfunction Questionnaire)

Scopus: 557 records

('dental health' OR 'oral healthcare' OR 'oral health' OR 'dental caries' OR 'periodontal health' OR 'periodontitis' OR 'oral health status' OR 'oral hygiene' OR 'oral health measures' OR 'cariogenic diet' OR 'toothbrushing' OR 'smoking' OR 'oral health barrier' OR 'oral health accessibility' OR ('oral health barrier' OR 'barriers to oral health') OR ('oral health accessibility' OR 'access to oral health')) AND ('Saudi Arabia' OR 'Kingdom of Saudi Arabia') AND ('cerebral palsy' OR 'autism' OR 'Asperger syndrome' OR 'Down syndrome' OR 'dyslexia' OR 'dyscalculia' OR 'dyspraxia' OR 'dysgraphia' OR 'blindness' OR 'visual impairment' OR 'deafness' OR 'hearing loss' OR 'hearing impairment' OR 'Attention deficit hyperactivity disorder' OR 'cystic fibrosis' OR 'learning disability' OR 'developmental disorder' OR 'learning disorder' OR 'intellectual disability' OR 'autism spectrum' OR 'hearing impaired') AND ('Oral Health-Related Quality of Life' OR 'Oral Health Impact Profile' OR 'General Oral Health Assessment Index' OR 'Oral Impacts on Daily Performance' OR 'Child-Oral

Impacts on Daily Performances' OR 'Early Childhood Oral Health Impact Scale' OR 'Child Perceptions Questionnaire' OR 'Parent-Caregiver Perceptions Questionnaire' OR 'Dental Impact on Daily Living' OR 'Oral Motor Dysfunction Questionnaire')

Google Scholar: 200 (limited to 20 pages)

(dental health OR oral healthcare OR oral health OR dental caries OR periodontal health OR periodontitis OR oral health status OR oral hygiene OR oral health measures OR cariogenic diet OR toothbrushing OR smoking OR oral health barrier OR oral health accessibility OR (oral health barrier OR barriers to oral health) OR (oral health accessibility OR access to oral health)) AND (Saudi Arabia OR Kingdom of Saudi Arabia) AND (cerebral palsy OR autism OR Asperger syndrome OR Down syndrome OR dyslexia OR dyscalculia OR dyspraxia OR dysgraphia OR blindness OR visual impairment OR deafness OR hearing loss OR hearing impairment OR Attention deficit hyperactivity disorder OR cystic fibrosis OR learning disability OR developmental disorder OR learning disorder OR intellectual disability OR autism spectrum OR hearing impaired) AND (Oral Health-Related Quality of Life OR Oral Health Impact Profile OR General Oral Health Assessment Index OR Oral Impacts on Daily Performance OR Child-Oral Impacts on Daily Performances OR Early Childhood Oral Health Impact Scale OR Child Perceptions Questionnaire OR Parent-Caregiver Perceptions Questionnaire OR Dental Impact on Daily Living OR Oral Motor Dysfunction Questionnaire)
